# Non-Invasive Electroanatomical Mapping: A State-Space Approach for Myocardial Current Density Estimation

**DOI:** 10.3390/bioengineering10121432

**Published:** 2023-12-16

**Authors:** Erik Engelhardt, Eric Elzenheimer, Johannes Hoffmann, Christy Meledeth, Norbert Frey, Gerhard Schmidt

**Affiliations:** 1 Department of Electrical Information Engineering, Faculty of Engineering, Kiel University, Kaiserstr. 2, 24143 Kiel, Germany; eren@tf.uni-kiel.de (E.E.); ee@tf.uni-kiel.de (E.E.);; 2 Internal Medicine 1—Cardiology and Internal Intensive Care Medicine, Med Campus III, Kepler University Hospital, Krankenhausstraße 9, 4021 Linz, Austria; christy.meledeth@kepleruniklinikum.at; 3 Department of Internal Medicine III (Cardiology, Angiology and Pneumonology), University Medical Center Heidelberg, Im Neuenheimer Feld 410, 69120 Heidelberg, Germany; norbert.frey@med.uni-heidelberg.de

**Keywords:** biomagnetism, magnetocardiography (MCG), noninvasive cardiac diagnostics, Kalman filter, gradient descent, optimization

## Abstract

Electroanatomical mapping is a method for creating a model of the electrophysiology of the human heart. Medical professionals routinely locate and ablate the site of origin of cardiac arrhythmias with invasive catheterization. Non-invasive localization takes the form of electrocardiographic (ECG) or magnetocardiographic (MCG) imaging, where the goal is to reconstruct the electrical activity of the human heart. Non-invasive alternatives to catheter electroanatomical mapping would reduce patients’ risks and open new venues for treatment planning and prevention. This work introduces a new system state-based method for estimating the electrical activity of the human heart from MCG measurements. Our model enables arbitrary propagation paths and velocities. A Kalman filter optimally estimates the current densities under the given measurements and model parameters. In an outer optimization loop, these model parameters are then optimized via gradient descent. This paper aims to establish the foundation for future research by providing a detailed mathematical explanation of the algorithm. We demonstrate the feasibility of our method through a simplified one-layer simulation. Our results show that the algorithm can learn the propagation paths from the magnetic measurements. A threshold-based segmentation into healthy and pathological tissue yields a DICE score of 0.84, a recall of 0.77, and a precision of 0.93.

## 1. Introduction

### 1.1. Motivation

Over the past century, our understanding of the human heart has increased dramatically. Today, we can diagnose and treat many more diseases than a hundred years ago. Despite this, heart disease remains the leading cause of death [1]. Cardiac arrhythmias may occur as a consequence of underlying heart disease such as cardiomyopathy or be a primary phenomenon such as in long QT syndrome, an inherited ion channel disorder. Accurately diagnosing arrhythmias often requires using invasive electroanatomical mapping techniques, thereby putting the patient at risk for complications such as bleeding or infection.

Non-invasive methods that provide the same result would spare them the risks associated with invasive measurements [2,3]. In combination with Stereotactic Body Radiation Therapy(SBRT) [4], this could potentially allow for completely non-invasive treatment of cardiac arrhythmias [5,6]. These methods would also allow measurements in cases where they are not medically indicated, providing an excellent opportunity for research and prevention.

### 1.2. State of the Art

The gold standard for electroanatomical mapping is invasive catheterization with systems such as EAMS, EnSite Velocity^®^, and CARTO^®^3. By tracking the position of the catheter and measuring the potential of the myocardium with the catheter head, a computer program reconstructs a map of the local activation time. Medical doctors can then use this information to select the region for ablation. To minimize risk and stress for the patients, mapping and ablation are usually performed during the same procedure. Therefore, an extensive analysis of the data prior to ablation is not possible. The invasive nature of this method also prevents its use as a diagnostic measurement. The reported accuracies of commercial systems are between 1mm and 2mm, while the reported threshold for clinical relevance is 3mm [7].

Non-invasive measurements aim to reconstruct electrical activity inside the myocardium indirectly from data such as electrocardiography (ECG), magnetocardiography (MCG) and magnetic resonance imaging (MRI). While the MRI data is used to construct a spatial model of the heart, the MCG or ECG data is used to reconstruct the electrical activity of the heart. The use of a patient-specific torso model is necessary to accurately calculate body surface potentials from myocardial currents [8,9,10]. Since the relative permeability of the human torso does not vary much, not including a torso model incurs fewer errors for forward magnetocardiographic models [11]. For this reason, we will focus on magnetic measurements [12], although an extension to include electric measurements might prove beneficial [11,13,14].

There are different approaches to solving the problem of non-invasive electroanatomical mapping. One way to circumvent the prohibitively large number of unknown parameters is to compute the current density distribution from a low-dimensional set of parameters. Gillette et al. [15] use a sophisticated electrophysiological model to compute the distribution of cardiac sources and ECG potentials over time. They then use parameter sampling to match the simulated ECG signals to the measurements. A major advantage of this digital twin approach is that the optimized parameters have well-understood electrophysiological meanings. The main limitation of this approach is the poor scaling of parameter sampling to higher-dimensional parameter vectors. Gillette et al. acknowledge that accurate modeling of substrate-based diseases would require regions of deviating parameters. At the opposite end of the spectrum from digital twins, there are supervised machine-learning approaches. Instead of fitting meaningful electrophysiological parameters to the measurements, models, usually deep neural networks, are trained to predict cardiac sources directly from non-invasive measurements. For example, Chen et al. [9] trained a convolutional neural network on pairs of heart and body surface potentials from five pigs. This approach is not limited to specific types of diseases, such as the cardiac digital twin approach, since no explicit assumptions about propagation pathways or mechanisms are made. The main challenge is acquiring suitable training data.

### 1.3. Contributions

In this paper, we present the mathematical foundation of a novel algorithm to estimate the current density distribution in myocardial tissue from MCG measurements. We demonstrate the capabilities and limitations using a simplified simulation. The medical problem we address primarily is the localization of arrhythmogenic tissue.

Similar to the cardiac digital twinapproach, the parameters of our model have an electrophysiological meaning. Unlike the cardiac digital twin approach, these parameters can be different for each voxel, allowing for inhomogeneities in the myocardial tissue. This is possible because we do not have to sample the parameter space. Intead, we optimize the model parameters using gradient descent. In contrast to supervised machine learning approaches, no access to heart surface potentials is required.

## 2. Methods

We divide the myocardium into $N_{v}$ equally sized voxels. For each voxel, the three-dimensional current density $j_{v}\left(n\right)=\left[j_{v,x}\left(n\right),j_{v,y}\left(n\right),j_{v,x}\left(n\right)\right]{}^{⊺}$ is tracked, with $n\in [0\ldots N_{m}-1]$, where $N_{m}$ is the number of considered time steps, and $v\in [0\ldots N_{v}-1]$ is the voxel index. The vector $j\left(n\right)=\left[j_{0}\left(n\right){}^{⊺},\ldots ,j_{N_{v}-1}\left(n\right){}^{⊺}\right]{}^{⊺}$ is the concatination of these current densities, and the matrix $J=\left[j\left(0\right),\ldots ,j\left(N_{m}-1\right)\right]{}^{⊺}$ is the vector of current densities over time. Similarly $Z=\left[z\left(0\right),\ldots ,z\left(N_{m}-1\right)\right]{}^{⊺}$ denotes all measurements over time. The measurement vector for one time step is written as $z\left(n\right)=\left[z_{0}\left(n\right),\ldots ,z_{N_{s}-1}\left(n\right)\right]{}^{⊺}$, where $N_{s}$ is the number of individual sensors. The measurements can be written as z(n)=d(n)+s(n), where d(n) is the desired signal and s(n) is the noise signal.

A forward model maps the estimated current densities $\hat{j}$ into the measurement space through a measurement matrix *H*, resulting in the estimated measurements $\hat{z}$:

(1) $$\hat{z}\left(n\right)=H\hat{j}\left(n\right).$$

Obtaining the estimated current densities by computing the pseudoinverse of the measurement matrix H and multiplying it by the measurements z is not feasible:

(2) $$\hat{j}\left(n\right)=H^{-1}z\left(n\right).$$

The reason for this is the ill-posed nature of the problem. Instead, the problem of non-invasive measurements is stated as finding the, in a sense, best estimate of J for some given measured signals Z:

(3) $$\hat{J}\leftarrow \underset{\hat{J}}{\arg\;\min}\;L\left(\hat{J},Z\right).$$

### 2.1. Overview

**Figure 1:**
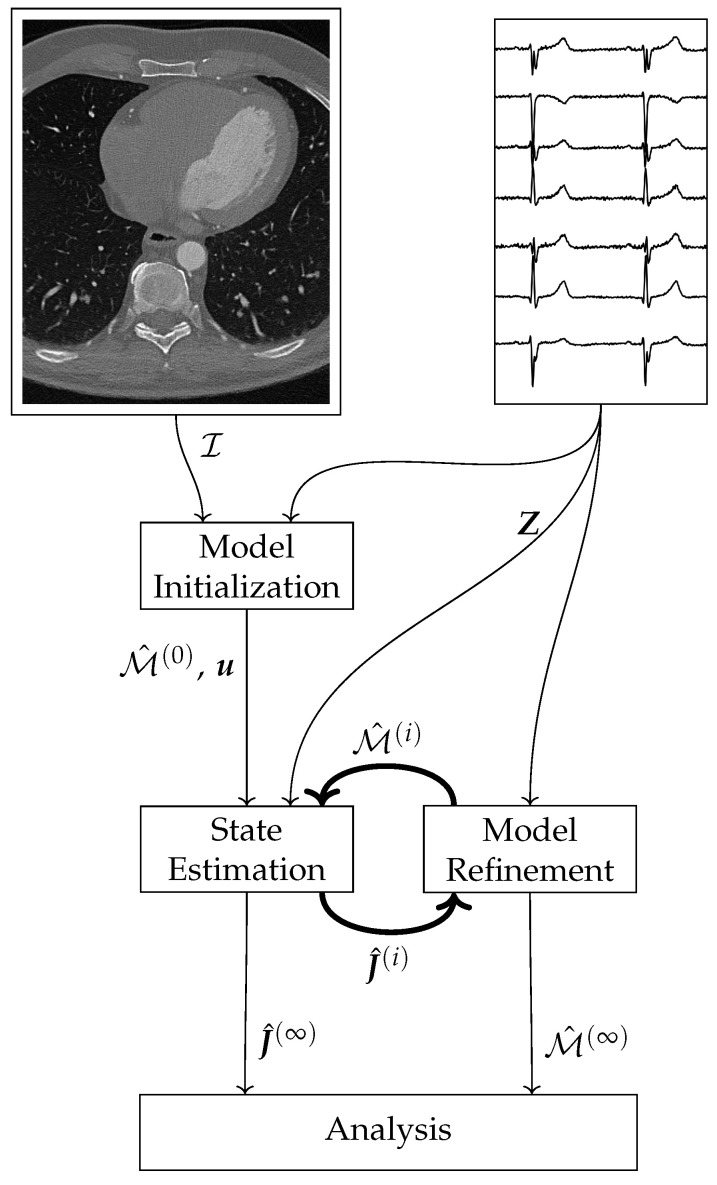
Schematic overview of the current density estimation algorithm.

The proposed current density estimation algorithm consists of three main parts: the Model Initialization, the State Estimation, and the Model Refinement (cf. Figure 1).

The Model Initialization uses image data I, and knowledge of the electrophysiology of a healthy heart to construct the initial state space model ${\hat{M}}^{\left(0\right)}$. In the State Estimation part, a sparse Kalman filter estimates the current densities ${\hat{J}}^{\left(i\right)}$ from the model ${\hat{M}}^{\left(i\right)}$ and the measurements Z, where the superscript *i* indicates the epoch. Using the estimated current densities ${\hat{J}}^{\left(i\right)}$ and the measurements Z, the new model ${\hat{M}}^{\left(i+1\right)}$ is calculated in the *Model Refinement* block. The main loop consists of the steps *State Estimation* and *Model Refinement*. These two steps execute alternately. After termination, the final estimated current densities ${\hat{J}}^{\left(\infty \right)}$ and the final model ${\hat{M}}^{\left(\infty \right)}$ can be used for further analysis.

### 2.2. Forward Model

The forward model describes the mapping of the current densities j into the measurements z. The measurement vector consists of $N_{s}$ magnetic measurements at positions $P_{s}=\left[p_{s,0}{}^{⊺},\ldots ,p_{s,N_{s}-1}{}^{⊺}\right]{}^{⊺}$ with orientations $O_{s}=\left[o_{s,0}{}^{⊺},\ldots ,o_{s,N_{s}-1}{}^{⊺}\right]{}^{⊺}$.We calculate the magnetic fields using a superposition of Biot-Savart’s law for all voxels:

(4) $$z_{i}\left(n\right)=\frac{\mu_{0}}{4\pi }\sum_{k=0}^{N_{v}-1}o_{s,i}{}^{⊺}j_{k}\left(n\right)\times \frac{p_{s,i}-p_{v,k}}{\left|\right|p_{s,i}-p_{v,k}{\left|\right|}_{2}^{3}}V,$$

where $N_{v}$ is the number of voxels $p_{v,k}$ is the position of the *k*-th voxel, and *V* is the volume of the voxels.

Using Equation (4) we calculate measurement matrix entries during the Model Initialization. In the State Estimation and Model Refinement step, the measurement matrix is used to calculate the estimated measurements by applying Equation (1).

### 2.3. System Model

The system model describes the prediction of system states j(n|n−1). The system model needs to facilitate arbitrary propagation paths and propagation velocities to accurately model the propagation of the action impulse through the myocardium [16,17,18]. Modeling the interactions between all voxels becomes intractable as the number of voxels increases. Therefore, we use First-order Thiran all-pass filters [19] to model the interaction between neighboring voxels.

These infinite impulse response (IIR) filters have an amplitude response of one over the whole frequency range and a constant group delay for $0\leq f≲f_{s}/10$. Within these limits, arbitrary group delays τ≥1sample can be achieved by applying the following equations:

(5) $$y\left(n\right)=a\;\cdot \;\left(\hat{j}\left(n-k-1\right)-y\left(n-1\right)\right)+\hat{j}\left(n-k-2\right).$$

Equation (5) is written for a single all-pass output *y*. The parameter *k* is the integer part of the desired group delay τ, while the parameter *a* determines the fractional part of the group delay [19]:

(6) $$a=\frac{1-\left(\tau \;\mathrm{mod}\;1\right)}{1+\left(\tau \;\mathrm{mod}\;1\right)}.$$

We calculate the predicted current densities by applying:

(7) $${\hat{j}}_{v}\left(n\right)=C_{v}y_{v}\left(n\right)+b_{v}u\left(n\right).$$

Here, ${\hat{j}}_{v}\left(n\right)$ is the three-dimensional current density in x-, y-, and z-direction in one voxel v. The vector $y_{v}\in R^{243}$ contains the all-pass outputs that contribute to $j_{v}$. The sparse matrix $C_{v}\in R^{3\times 243}$ contains the 243 gains *c*, that map the all-pass outputs into the current densities. The influence of the control function u(n) on the current density ${\hat{j}}_{v}\left(n\right)$ is modelled by the corresponding entry in the control vector $b_{v}$.

The control function determines the shape of the action potential. We use *Myokit* [20] to calculate an action potential based on the dynamic O’Hara-Rudy model [21]. The control function u(n) is calculated by differentiating the action potential and scaling it to a maximum value of 1 A/mm^2^.

### 2.4. Model Initialization

In the Model Initialization, the initial values for the delays τ (cf. Equations (5) and (6)), the gains c (cf. Equation (7)), and the control vector b are computed based on the voxel positions $P_{v}=\left[p_{v,0}{}^{⊺},\ldots ,p_{v,N_{v}-1}{}^{⊺}\right]$ and the voxel types $\zeta =\left[\zeta_{0},\ldots ,\zeta_{N_{v}-1}\right]{}^{⊺}$.

**Figure 2:**
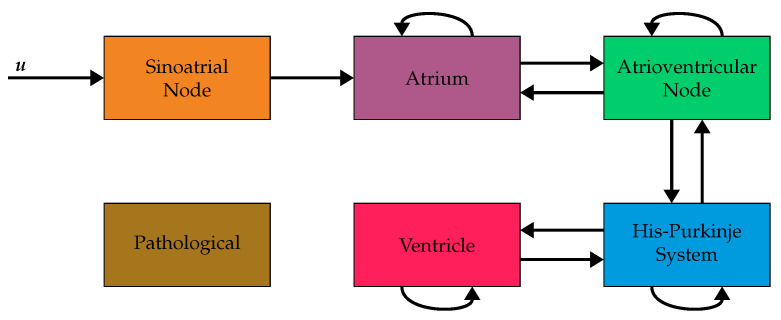
Block diagram of the assumed connectivities between the six considered voxel types and the input function u. The input function u is connected to the sinoatrial node. The other arrows indicate allowed connections between neighboring voxels. For example, a direct connection from atrium to ventricleis not allowed. Instead, the propagation has to pass through the atrioventricular node and the HIS-Purkinje system before reaching the ventricles. Voxels of type pathological are not connected to other voxels in this model and, therefore, never excited. (Figure adapted from [24]).

We consider six voxel types ζ in this study: sinoatrial node, atrium, atrioventricular node, His-Purkinje system, ventricle, and pathological. These six types are the minimum number needed to model the electrophysiology of the heart sensibly. They allow for a localized excitation of the heart, a localized connection between the atria and ventricles, a coordinated excitation of the ventricles via the His-Purkinje system, and a disturbed function via the pathological voxels. The assumed connectivities between the voxel types [15,18,22,23] are shown in Figure 2.

First, we calculate the delays $\tau^{\left(0\right)}$ for all pairs of neighboring voxels based on their positions p and the propagation velocity ϕ of the input voxel type $\zeta_{i}$. For all voxels $v_{i},i\in \left[0\ldots ,N_{v}-1\right]$ and their up to 26 neighbors $v_{o}$, the delay $\tau_{i,o}^{\left(0\right)}$ is calculated according to:

(8) $$\tau_{i,o}^{\left(0\right)}=\frac{\left|\right|p_{i}-p_{o}{\left|\right|}_{2}}{\phi (\zeta_{i})}\;\cdot \;f_{s}.$$

The variable $\tau_{i,o}^{\left(0\right)}$ describes the delay of the propagation from voxel $v_{o}$ to voxel $v_{i}$ in samples.

Next, the gains C are calculated using an iterative algorithm. We use the symbol $C_{i,o}\in R^{3\times 3}$ to denote the nine gain entries that describe the propagation from voxel $v_{o}$ to voxel $v_{i}$. It is necessary to keep track of the activation times of each voxel. We denote these as $\sigma \in R^{N_{v}}$. Furthermore, it is necessary to keep track of the current directions in each voxel. We denote these as $\delta \in R^{N_{v}\times 3}$. We initialize the activation time of the sinoatrial node with $\sigma_{\mathrm{sinoatrial}}=0\;\mathrm{ms}$ and calculate the others during the iterative procedure detailed below. This ensures that the activation in the initial model starts in the sinoatrial node. We arbitrarily choose the current direction of the sinoatrial node to be $\delta_{\mathrm{sinoatrial}}=\left[1,0,0\right]{}^{⊺}$. The entries of the gain matrix C are calculated by the following procedure.

1. The iteration time $t_{i}$ is set to 0 ms.
2. For all voxels $v_{o}$ with an activation time $\sigma_{o}$ equal to the iteration time $t_{i}$ the following steps are carried out.
  (a) Identify all neighboring voxels $v_{i}$, that have not been connected yet.
  (b) Discard all neighbors with incompatible voxel types (cf. Figure 2).
  (c) Calculate the current direction in $v_{i}$ according to the following equation, where $p_{i}$ is the position of the voxel $v_{i}$ and $p_{o}$ it the position of the voxel $v_{o}$:
    (9) $$\delta_{i}=\frac{p_{i}-p_{o}}{\left|\right|p_{i}-p_{o}{\left|\right|}_{1}}.$$
  (d) Calculate the gains $C_{i,o}$ between two voxels $v_{i}$ and $v_{o}$ according to the following equation:
    (10) $$c_{j,k}=\delta_{i,j}\;\cdot \;\mathrm{sign}\left(\delta_{o,k}\right).$$
    The indices *i* and *o* are dropped for better readability and the indices j,k∈{0,1,2} are used to index the three by three matrix $C_{i,o}$.
  (e) Calculate the activation time of the voxel $v_{i}$ as $\sigma_{i}=\sigma_{o}+\tau_{i,o}\;\cdot \;T_{s}$.
3. Set the iteration time $t_{i}$ to the smallest activation time σ that is bigger than the current iteration time $t_{i}$.

We repeat steps two and three until we do not find a new activation time in step three. This procedure ensures that fast propagation paths are preferred, no voxel is activated twice, and all voxels that can be connected are connected.

Since the control function is scalar, the control matrix B simplifies to a vector $b\in R^{3N_{v}}$. We set the entry corresponding to the x component of the current density of the sinoatrial node to one and all others to zero.

### 2.5. State Estimation

The *State Estimation* uses a sparse Kalman filter to estimate the current densities ${\hat{J}}^{\left(i\right)}$ using the current model ${\hat{M}}^{\left(i\right)}$ and the measurements Z. For each timestep, we carry out the following steps. First, we predict the current densities $\hat{j}\left(n|n-1\right)$ by applying Equations (5) and (7). Next, we predict the state covariance matrix $\hat{P}\left(n|n-1\right)$ by applying:

(11) $$p_{i,o}=q_{i,o}+\sum_{k\in N_{o}}c_{o,k}\sum_{m\in N_{i}}c_{i,m}p_{m,k}\;,o\in N_{i}.$$

Since the gains *c* are defined only for neighboring voxels, the summations include only said neighbors. We denote this with N. Then, the Kalman gain K(n) is computed by applying:

(12) $$s_{i,o}=r_{i,o}+\sum_{k=0}^{3N_{v}-1}h_{o,k}\sum_{m\in N_{k}}h_{i,m}p_{m,k},$$

(13) $$k_{i,o}=\sum_{k=0}^{N_{s}-1}s_{k,o}^{-1}\sum_{m\in N_{i}}p_{i,m}h_{k,m}.$$

We use $s_{m,o}^{-1}$ to denote the entry (m,o) of the inverse $S^{-1}$. After that, we correct the current densities $\hat{j}\left(n|n\right)$ using the measurements z(n) by applying:

(14) $$\hat{j}\left(n|n\right)=\hat{j}\left(n|n-1\right)+K\left(z\left(n\right)-\hat{z}\left(n|n-1\right)\right)$$

Finally, we correct the state covariance matrix $\hat{P}\left(n|n\right)$ using the measurements z(n) by applying:

(15) $$p_{i,o}=\sum_{k\in N_{o}}p_{k,o}\left(i_{i,k}-\sum_{m=0}^{N_{s}-1}k_{i,m}h_{m,k}\right)\;,o\in N_{i}.$$

### 2.6. Model Refinement

The Model Refinement uses the estimations of the current densities ${\hat{J}}^{\left(i\right)}$ and the measurements Z to adjust the model $M^{\left(i\right)}$, yielding the refined model $M^{\left(i+1\right)}$. To this end, we calculate a loss function L(n) for each time step. Based on this loss we calculate the gradients $\frac{\partial L}{\partial c}$ and $\frac{\partial L}{\partial a}$. We then adjust the gains *c* and the all-pass coefficients *a* using these gradients. The goal is to minimize the difference between the actual current densities J and the estimated current densities $\hat{J}$. Since we do not have access to the actual current densities J we minimize the difference between ground truth measurements Z and predicted measurements $\hat{Z}$ instead. We denote this part of the loss as $L_{m}$:

(16) $$L_{m}=\frac{1}{N_{s}}\sum_{k=0}^{N_{s}-1}{\left(z_{k}-{\hat{z}}_{k}\right)}^{2}.$$

We place an electrophysiologically motivated constraint on the estimated current densities to restain the possible solutions. The absolute current density in a voxel may be lower than the one imposed by the control function u(n) due to the voxel not being fully filled with myocardial tissue or changes of the myocardial tissue filling the voxel [16,25,26]. There is, however, no reason for the current density to exceed the nominal maximum value. Therefore, we add a term to the loss that increases once the absolute current density in a voxel $\left|\right|{\hat{j}}_{v}{\left|\right|}_{1}$ exceeds the normalized value of 1.01, and is zero otherwise. We denote this part of the loss as $L_{v}$:

(17) $$L_{v}=\sum_{v=0}^{N_{v}-1}\max\{0,(||{\hat{j}}_{v}{\left|\right|}_{1}-1.01{)}^{2}\}.$$

The final loss is the sum of both parts weighted by the regularization strength γ:

(18) $$L=L_{m}+\gamma L_{v}.$$

In order to update the gains *c* and delays τ, we have to calculate the respective partial derivatives of the loss function. Starting with $\frac{\partial L_{m}}{\partial c}$ and applying the chain rule:

(19) $$\frac{\partial L_{m}}{\partial c}=\frac{\partial j}{\partial c}\frac{\partial \hat{z}}{\partial j}\frac{\partial L_{m}}{\partial \hat{z}},$$

we arrive at:

(20) $$\frac{\partial L_{m}}{\partial c}=yh^{⊺}\frac{2}{N_{s}}\left(\hat{z}-z\right),$$

where *y* is the all-pass output corresponding to the gain *c* and h is the column of the measurement matrix H corresponding to the current density *j* influenced by the gain *c* (cf. Equation (7)). Next, $\frac{\partial L_{v}}{\partial c}$ evaluates to:

(21) $$\frac{\partial L_{m}}{\partial c}=2\;\cdot \;y\;\cdot \;\mathrm{sign}\left(\hat{j}\right)\;\cdot \;\max\{0,||{\hat{j}}_{v}{\left|\right|}_{1}-1.01\}$$

where *y* is the all pass output corresponding to *c*, $\hat{j}$ is the estimated current density corresponding to *c*, and ${\hat{j}}_{v}$ are the three current densities, including $\hat{j}$ corresponding to the voxel influenced by *c*. For $\frac{\partial L_{m}}{\partial a}$ we can again apply the chain rule:

(22) $$\frac{\partial L_{m}}{\partial a}=\sum_{y}\frac{\partial y}{\partial a}\frac{\partial j}{\partial y}\frac{\partial \hat{z}}{\partial j}\frac{\partial L_{m}}{\partial \hat{z}},$$

arriving at:

(23) $$\frac{\partial L_{m}}{\partial a}=\sum_{y}\frac{\partial y}{\partial a}ch_{y}^{⊺}\frac{2}{N_{s}}\left(\hat{z}-z\right).$$

The summation over the corresponding all-pass outputs *y* is because the all-pass coefficients are shared across nine all-pass filters that connect two voxels. Calculating $\frac{\partial y}{\partial a}$ is done by applying the following iterative equations:

(24) $$\frac{\partial y}{\partial a_{\mathrm{IIR}}}\left(n\right)=y\left(n-1\right)+a\frac{\partial y}{\partial a_{\mathrm{IIR}}}\left(n-1\right),$$

(25) $$\frac{\partial y}{\partial a_{\mathrm{FIR}}}\left(n\right)=j\left(n-k-1\right)+a\frac{\partial y}{\partial a_{\mathrm{FIR}}}\left(n-1\right),$$

where $\frac{\partial y}{\partial a}=\frac{\partial y}{\partial a_{\mathrm{IIR}}}+\frac{\partial y}{\partial a_{\mathrm{FIR}}}$ ([27], Chapter 15). Lastly, since the parameters τ only influence the propagation velocity, the regularization loss $L_{v}$ is not used in the calculation of $\frac{\partial L}{\partial a}$. In other words, $\frac{\partial L_{v}}{\partial a}$ is set to zero.

Using these gradients we then update the gains *c* according to:

(26) $$c^{\left(i+1\right)}=c^{\left(i\right)}-\frac{\eta }{N_{m}}\sum_{n=0}^{N_{m}-1}\frac{\partial L^{\left(i\right)}}{\partial c^{\left(i\right)}}\left(n\right)$$

and the all-pass coefficients according to

(27) $$a^{\left(i+1\right)}=a^{\left(i\right)}-\frac{\eta }{N_{m}}\sum_{n=0}^{N_{m}-1}\frac{\partial L^{\left(i\right)}}{\partial a^{\left(i\right)}}\left(n\right),$$

where η is the learning rate.

The all-pass coefficient *a* is only valid for values between zero and one. If, after applying Equation (27), the coefficient *a* is less than zero, it is increased by one, and the integer part of the delay (cf. Equation (5)) is also increased by one. If, on the other hand, the coefficient exceeds one, we have to consider two cases. If the integer part of the delay is at least one, we reduce the coefficient and the integer part by one. Otherwise, we set the coefficient is set to one.

## 3. Simulations

The question we want to answer with the following simulations is if our state-space current density estimation approach is able to localize regions of reduced electrical activity, which would indicate the presence of arrhythmogenic tissue [28]. While our approach can learn propagation paths and velocities from magnetic measurements, we focus on the propagation paths in these simulations. The comparison with other sophisticated approaches, as introduced in Section 1.2, remains challenging due to the lack of available source code. Therefore, we compare our results to the pseudoinverse solution (cf. Equation (2)) instead. The current density estimation should yield results that facilitate differentiation between healthy and pathological tissue. Therefore, we employ a simple threshold-based segmentation.

### 3.1. Setup

**Figure 3:**
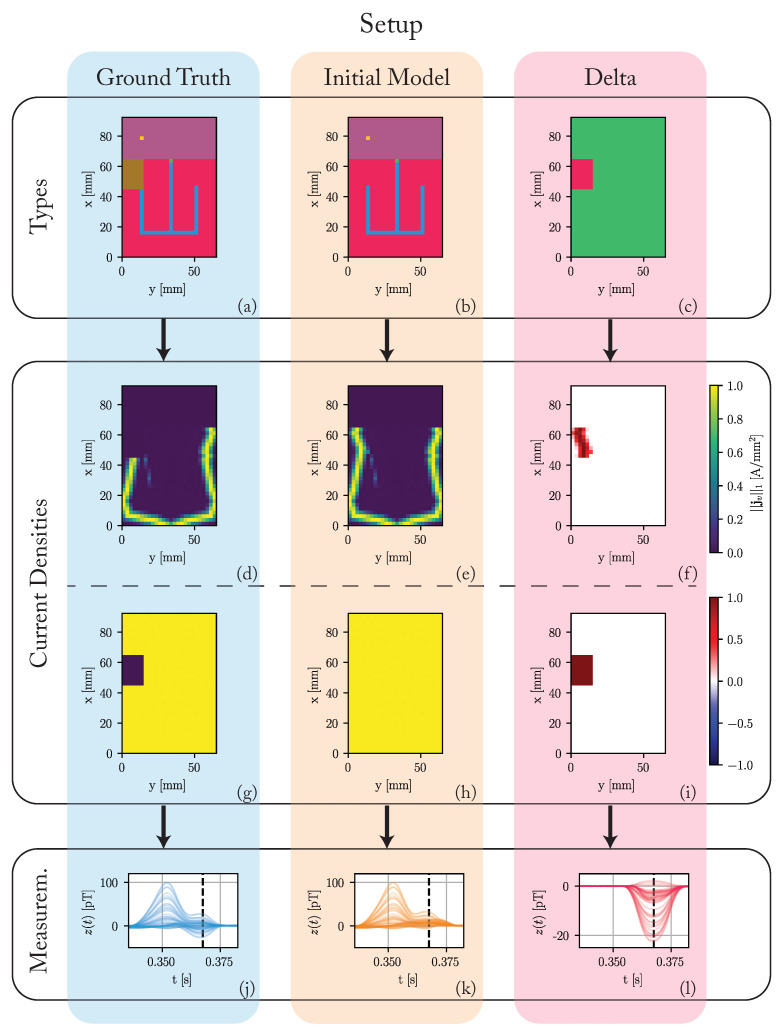
Overview of the used simulation setup. (**a**) Voxel types of the ground truth model: The colors of the voxels correspond to their type as depicted in Figure 2. A region of pathological tissue is present in the right ventricle. (**b**) Voxel types of the initial model: The initial model assumes a healthy heart without any pathological regions. (**c**) The difference in voxel types: red regions stand for differences between the ground truth and initial model. Green regions stand for equalities. (**d**–**i**) The L1-norm of the current densities in each voxel. (**d**–**f**) During the time of the maximal difference between ground truth and initial model (t=0.3675 s). (**g**–**i**) The maximum of this L1-norm over the complete cardiac cycle. (**j**–**l**) The simulated magnetic fields *z* around the time of the QRS-complex. The black dashed line corresponds to the time of the maximum difference between the ground truth and the initial model (t=0.3675 s).

To answer this question, we use an abstracted one-layer heart simulation as depicted in Figure 3. The heart model consists 25×37×1 (x, y, z) voxels of size 2.5 mm, resulting in a total heart size of 65 mm×92.5 mm×2.5 mm (cf. Figure 3a). This model represents the unrolled surface of the heart. The voxel types are assigned based on the approximate shape of the regions in the real heart to generate a morphologically sound sinus rhythm. We differentiate between the ground truth model M, used to generate the data, and the initial model ${\hat{M}}^{\left(0\right)}$. While the initial model assumes a healthy heart without any pathological regions, the ground truth model includes a region without any conduction in the right ventricle. We show the difference in voxel types between ground truth and the initial model in Figure 3c. The assumed propagation velocities are 1.1 m/s for the sinoatrial node, atrium, and ventricle, 0.012 m/s for the atrioventricular node, and 4.5 m/s for the HIS-Purkinje system. While these values are simplified, we based them on values reported in literature [15,18,22,23]. We run both models for a simulation time of 1 s with a simulation frequency of 2000 Hz.

Figure 3d,e depict the L1-norm of the simulated current densities in each voxel for t=0.3675 s. At this time, the propagation already passed through the HIS-Purkinje system and is spreading outwards through the ventricles. The propagation is symmetrical for the initial model, while the ground truth has no electrical activity in the pathological region. Figure 3f shows the difference in these L1-norms. The current density in the initial model is higher than in the ground truth model in the region of the pathology. Since we cannot show all time steps in this fashion, Figure 3g–i show the maximum over these L1-norms during the complete cardiac cycle. While this maximum is constant over all voxels in the initial model, the ground truth model has no electrical activity in the pathological region for the complete cardiac cycle.

We use a 4×4×3 array of ideal magnetic sensors. The sensors are placed equally across a 250mm×250mm×100mm region centered 200 mm above the heart. Since we simulate one layer of voxels, the measurements have to be scaled up to match reasonable field amplitudes as reported in [29,30,31]. We scale the measurements by a fixed factor of 70 to achieve a maximum R-peak amplitude of 100 pT for the ground truth model. We then superimpose the simulated signals with white noise of an equivalent noise spectral density of 40 fT/$\sqrt{\mathrm{Hz}}$, which is in the order of low temperature superconducting quantum interference devices (SQUIDs) [8]. Figure 3j–l depict the magnetic measurements around the time of the QRS-complex. The dashed black line indicates the time of the maximum difference (22.5 pT) between the ground truth and the initial model at time t=0.3675 s.

The number of voxels is $N_{v}=962$, resulting in 2886 system states. The number of sensors is $N_{s}=144$, which is on the high end in the number of sensors of available MCG systems [29].

### 3.2. Results

#### 3.2.1. Pseudoinverse

**Figure 4:**
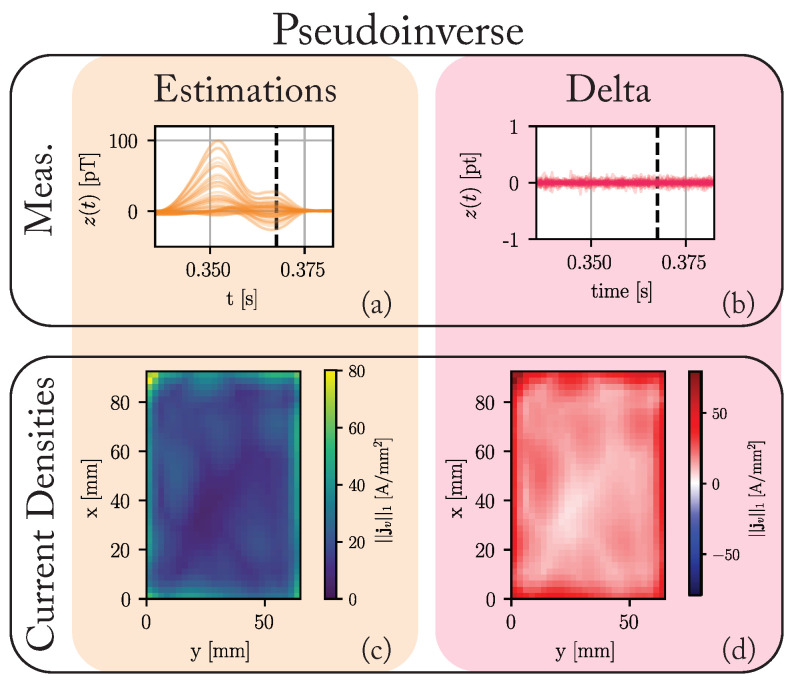
The results of applying the pseudoinverse solution to the data generated by the ground truth model introduced in Figure 3. (**a**) The estimated measurements in pT. (**b**) The difference between the predicted measurements and the ground truth measurements (cf. Figure 3j). (**c**) Maximum of the L1-norm of the current densities in each voxel over the complete cardiac cycle. (**d**) The difference between these maxima and those of the ground truth (cf. Figure 3g).

Figure 4 summarizes the results of the pseudoinverse solution. Since the number of unknown values greatly exceeds the number of knowns, the differences between the predicted measurements and the ground truth are vanishingly small. The same, however, can not be said for the current densities. Here, no discernable structure is present. This solution favors using the outer voxels to reconstruct the magnetic measurement, but no segmentation into healthy and pathological tissue is possible based on these results.

#### 3.2.2. State-Space Approach

**Figure 5:**
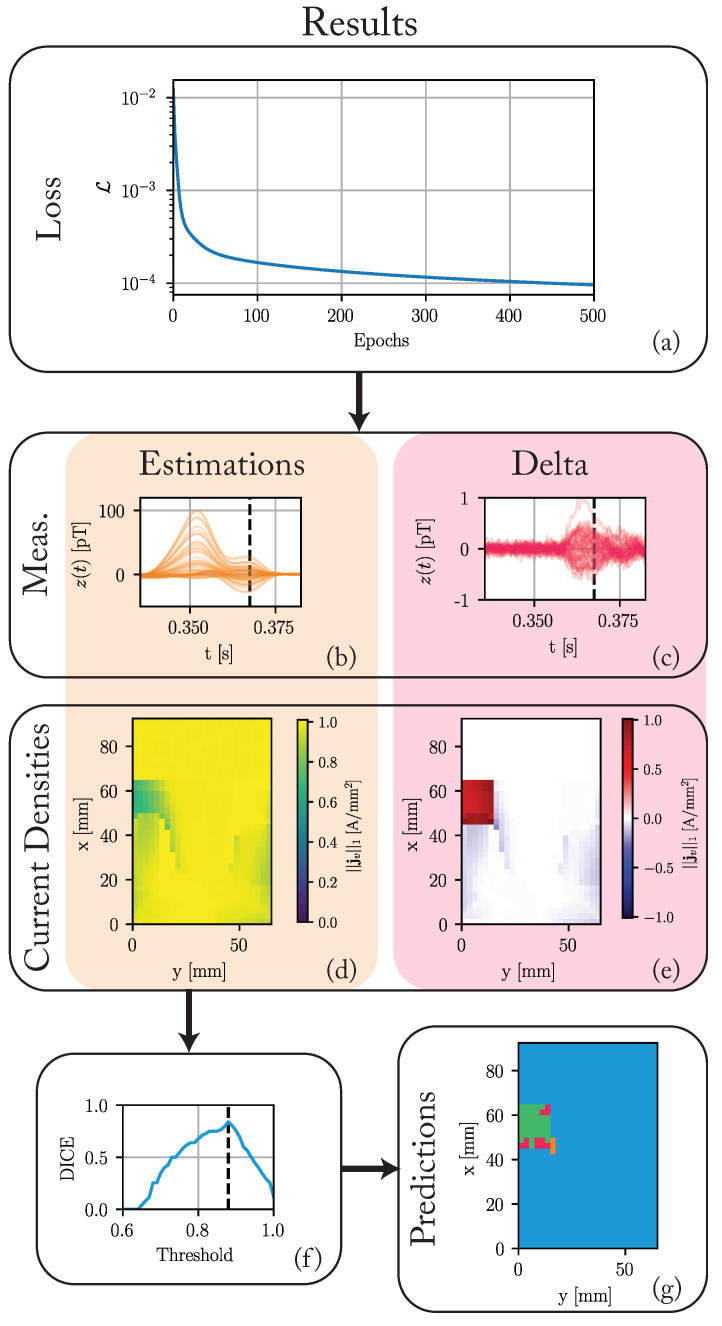
The results of the nested state-space optimization procedure after 2000 epochs. (**a**) The loss over the first 500 epochs. (**b**) The estimated magnetic measurements in pT. (**c**) The difference between these estimates and the ground truth (cf. Figure 3j). (**d**) Maximum of the L1-norm of the current densities in each voxel throughout the cardiac cycle. (**e**) The difference between these maxima and those of the ground truth (cf. Figure 3g). (**f**) The DICE score for different segmentation thresholds. A threshold of 0.88 achieves the maximum DICE score of 0.84. (**g**) The predicted voxel types for a threshold of 0.88. The color-coded regions stand for: green—true positive (TP), blue—true negatives (TN), orange—false positive (FP), and red—false negatives (FN).

Figure 5 summarizes the results of the state-space approach. We optimized the model for 2000 epochs with a learning rate of η=200 and a regularization strength of γ=1.

Figure 5a shows the loss function for the first 500 epochs. Although the loss decreases slightly until the last epoch to a final value of 5.8 × 10^−5^, the maximum decrease happens in the earlier epochs. The maximum of the measurement differences decreases significantly from around 20 pT to 1 pT. The differences in current densities to the ground truth in pathological tissue regions are significantly reduced, while differences in healthy regions increase slightly.

Figure 5f,g show the results of the simple threshold-based segmentation. The segmentation compares the maximum L1-norm in each voxel to a threshold. We classify the voxel as pathological if the L1-norm is below a threshold. Otherwise, we classify it as healthy. Based on this segmentation, a DICE score is calculated. Figure 5f shows this DICE score for all thresholds from 0.6 to 1.0. A threshold of 0.88 achieves the maximum DICE score of 0.84. Figure 5g shows the results of a segmentation based on this optimal threshold. Most pathological voxels are correctly classified. We achieve a recall of 0.77. Not many healthy voxels are incorrectly classified as pathological, achieving a precision of 0.93. All false positives (FP) are adjacent to the actual pathological region.

Executing one epoch of this algorithm takes around 1 s on an M2 MAX processor, using a single core. The algorithm is mostly converged after 500 epochs or 8 min. All 2000 epochs take around 30 min.

### 3.3. Discussion

We simulated a single layer of tissue leading to a relatively small number of voxels. At the same time, the number of sensors is on the high end for MCG sensor systems. Even in these favorable circumstances, the naive solution of using the pseudoinverse is not feasible. More sophisticated approaches are needed to extract useful information about the current density distribution in the human heart from non-invasive measurements.

While our state-space approach is not able to perfectly reconstruct the ground-truth current densities, the results show a clear structure. Throughout the optimization, the difference between the estimated magnetic measurements and the ground-truth measurements is reduced. The same holds for the current densities. The optimized model shows a reduced maximum current density in the pathological region of the ground truth model. For a perfect reconstruction, the maximum current density in this region would have to be zero. The optimized model only reduces the maximum to around 0.5A/mm^2^ in this region. At the same time, the maximum current density is also slightly reduced in regions that do not correspond to the pathological region in the ground truth model. The most obvious reason for this is that while our state-space approach applies a strong regularization and bias on the estimated current densities, the ill-posedness of the problem still leads to non-unique solutions. An extensive hyperparameter optimization over the learning rate, regularization strength, and Kalman parameters is expected to improve these results. With a DICE score of 0.84, segmentation solely based on the maximum current densities in each voxel is possible. These findings prove, that it is possible to differentiate between pathological and healthy tissue for this simplified model. This does suggest that regions with reduced electrical activity could also be found in real hearts, given sufficient measurements.

There are currently several limitations to our approach. First, we do not explicitly model any secondary currents caused by return paths. The update step of the State Estimation block implicitly calculates the return currents that flow through the myocardium. Our model cannot describe currents outside these voxels. Second, magnetic sensors do not measure the magnetic field perfectly. They only have a limited linear region and a non-flat frequency response [29]. Furthermore, they do not measure the magnetic field at one point but accumulate it over their sensing area. Third, there is a gradient in the refractory period in the myocardium [32]. Our model cannot capture this aspect of the electrophysiology of the heart. The control function encodes the de- and repolarization phases of the action potential. Since both are propagated using the same parameters, the refractory time cannot be varied depending on the location of the voxel. Since the signal generated by the depolarization phase is dominant over the one generated by the repolarization phase, the expected impact on the localization of arrhythmogenic tissue is low.

In order to apply this algorithm to patient data the following steps have to be taken. First, generating the spatial model of the patient’s heart requires a segmented MRI scan. Manual segmentation is feasible for proof of concept studies. For widespread application, automatic segmentation [33] is needed to reduce the time effort. Second, the initial propagation velocities can be estimated using characteristic times of the measured MCG. For example, researchers can use the P-wave’s duration and the spatial extent of the atrium to calculate the average propagation velocity in this area. After this, one can apply the algorithm as outlined in this paper. The resulting estimated current densities can then inform the decisions of medical professionals. With an execution time of 30 min, the algorithm is viable in clinical practice. The execution time will increase when applied to a whole heart instead of just one layer. There are, however, still opportunities to speed up the algorithm by using multithreading or fine-tuned code optimization. Another practical concern is that body movements (mainly respiration-induced) cause a subtle time-varying position change of the sensor array towards the heart. However, magnetic localization approaches (as conceptualized in [34]) can provide the position data required to compensate for such artifacts. While most clinics have access to MRI systems, they rarely have access to MCG systems [35]. An extension of our algorithm to also work for the more readily available ECG data would necessitate the extension of the forward model, including the consideration of the torso geometry and permittivities [8,9,10].

## 4. Conclusions

This paper introduces a novel state-space algorithm for the non-invasive estimation of cardiac current densities from MCG signals, allowing for arbitrary propagation paths and velocities. We demonstrated the algorithm’s ability to differentiate between healthy and pathological tissue in a simulated environment. Segmentation with the optimal threshold achieves a DICE score of 0.84. The presented results prove the ability to learn propagation paths from magnetic measurements. An extension of the algorithm to deal with the current limitation and apply it to patient data is feasible. Potentially, the algorithm can non-invasively localize arrhythmogenic tissue in clinical settings after further development.

## Data Availability

The data presented in this study are available on request from the corresponding author.

## Abbreviations

The following abbreviations are used in this manuscript:

AV	Atrioventricular
EEG	Electroencephalography
ECG	Electrocardiography
HPS	HIS-Purkinje system
MCG	Magnetocardiography
MRI	Magnetic resonance imaging
SA	Sinoatrial
SBRT	Stereotactic Body Radiation Therapy
SQUID	Superconducting quantum interference device
